# Aptamers: A New Technological Platform in Cancer Immunotherapy

**DOI:** 10.3390/ph9040064

**Published:** 2016-10-24

**Authors:** Fernando Pastor

**Affiliations:** 1 Instituto de Investigación Sanitaria de Navarra (IDISNA), Recinto de Complejo Hospitalario de Navarra, Pamplona 31008, Spain; 2 Program of Molecular Therapies, Aptamer Unit, Centro de Investigación Medica Aplicada (CIMA), Pamplona 31008, Spain; fpasrodri@unav.es; Tel.: +34-948-19-47-00

**Keywords:** aptamer, cancer immunotherapy, costimulation, immune-checkpoints

## Abstract

The renaissance of cancer immunotherapy is, nowadays, a reality. In the near future, it will be very likely among the first-line treatments for cancer patients. There are several different approaches to modulate the immune system to fight against tumor maladies but, so far, monoclonal antibodies may currently be the most successful immuno-tools used to that end. The number of ongoing clinical trials with monoclonal antibodies has been increasing exponentially over the last few years upon the Food and Drug Administration (FDA) approval of the first immune-checkpoint blockade antibodies. In spite of the proved antitumor effect of these reagents, the unleashing of the immune system to fight cancer cells has a cost, namely auto-inflammatory toxicity. Additionally, only a small fraction of all patients treated with immune-checkpoint antibodies have a clinical benefit. Taking into account all this, it is urgent new therapeutic reagents are developed with a contained toxicity that could facilitate the combination of different immune-modulating pathways to broaden the antitumor effect in most cancer patients. Based on preclinical data, oligonucleotide aptamers could fulfill this need. Aptamers have not only been successfully used as antagonists of immune-checkpoint receptors, but also as agonists of immunostimulatory receptors in cancer immunotherapy. The simplicity of aptamers to be engineered for the specific delivery of different types of cargos to tumor cells and immune cells so as to harvest an efficient antitumor immune response gives aptamers a significant advantage over antibodies. In this review all of the recent applications of aptamers in cancer immunotherapy will be described.

## 1. Introduction

The recent success in clinical trials of different approaches to elicit protective immunity in cancer patients, especially immune-checkpoint blockade antibodies, have brought to prominence the importance of the immune system in the control of tumors [1,2,3]. Cancer immunotherapy has become a reality and currently belongs to the therapeutic arsenal in oncology, even rivaling other standard treatments [4]. Nowadays, the most extended therapeutic agents are monoclonal antibodies, showing unprecedented clinical responses in advanced cancer patients. However, there are still some important limitations. Although the patients that respond to the immune-checkpoint blockade antibodies are long-lasting survivors (free of disease), the amount of responders is quite modest; additionally, the treatment is not completely devoid of toxicity [2]. In some cases, severe auto-inflammatory responses are observed, precluding the continuation of the treatment [5]. In order to increase the efficacy of cancer immunotherapy, all preclinical evidence indicates that multipronged approaches using a combo of different immune-modulatory agents need to be taken [6,7]. Inevitably, the use of several immunostimulatory agents would increase the chances of eliciting severe autoimmune-like responses [7]. Therefore, it is urgent to generate target therapeutic agents with fewer side effects than monoclonal antibodies. To this end, and based on recent preclinical studies, oligonucleotide aptamers may fit that chore very well.

## 2. Oligonucleotide Targeting Aptamers

There is evidence in nature that nucleic acids, especially RNA, can fold into complex globular structures with protein-like functions. In fact, the ribosome, one of the most complex enzymes found in nature and responsible for protein translation, is made of RNA. Thus, it is reasonable to consider the feasibility to select high-affinity and specific oligonucleotide ligands that exhibit complex globular tertiary structures. The term “aptamer” was coined by Elligton and is derived from the Latin word “aptus,” which means “fit,” and the Greek word “meros,” which means “particle.” The first oligonucleotide aptamers were described several years ago by Elligton and Gold [8,9]. They were selected throughout a complex combinatorial interactive technique named Systematic Evolution of Ligands by Exponential Enrichment (SELEX). This is a very powerful purification method, allowing for the isolation of rare molecules from an extremely large, complex library. The SELEX method can be divided into three major steps (binding, partition, and amplification). Several variants of SELEX have been described (further information on this topic is available in [10]). Probably one of the most valuable recent modifications of conventional SELEX is the introduction of high-throughput sequencing technology. Deep sequencing, together with new IT packages, allows for the identification of high-affinity aptamers in early rounds of selection, thus reducing the time and cost of selection, but, most importantly, minimizing the introduction of undesirable bias accumulated along the selection process in the amplification step, which is carried out by Polymerase Chain Reaction (PCR) [11,12,13,14]. The nature of the aptamer-oligonucleotide backbone can be made of DNA or RNA, and there are several modifications available that can be used to increase the stability of the aptamer in serum, by substitution of ribonucleotides with 2′-amino, 2′-fluoro, or 2′-*O*-alkyl nucleotides, among others [15]. Some research groups are using nucleotide analogs in order to improve the affinity and specificity of the aptamer, supposedly increasing the number of different types of ribonucleotides, which could increase the aptamer structural complexity [16,17]. This may be of great importance for targets with acidic isoelectric points that are negatively charged. In these cases, the introduction of base analogs of neutral or positive charge might favor the interaction with the negatively-charged target.

Oligonucleotide-derived aptamers display some important advantages when compared with monoclonal antibodies. Monoclonal antibodies, as cell-based products, have a much higher production cost and a complex regulatory approval to reach good manufacturing production (GMP), whereas aptamers are chemically-derived products, which implies a reduced production cost. As mentioned before, cancer immunotherapy is leading towards multipronged approaches aimed at combining several immune-modulatory reagents. Therefore, it would be desirable to have simpler regulatory approval and more cost-effective translational reagents to combine them in future clinical trials. Protein molecules, as monoclonal antibodies, are more likely to induce T cell-dependent neutralizing antibodies after repeated administrations that could be critical in long-lasting treatments of chronic diseases. In cancer patients that have been treated with immune-checkpoint monoclonal antibodies there is still no data on this mechanism of resistance, but it should be taken into consideration for study as a possible mechanism of resistance, especially in responder patients that relapse and need to reinitiate treatment. The presence of neutralizing antibodies that reduces or abrogates the therapeutic efficacy has been documented even in whole humanized monoclonal antibodies [17]. So far, and to the best of my knowledge, there is no evidence of induction of neutralizing antibodies against any therapeutic aptamer. Probably one of the most important advantages of aptamers is managing side effects associated with immune-modulatory agents linked to exacerbated auto-inflammatory immune responses. Monoclonal antibodies have shown severe side effects in cancer immunotherapy [18,19,20,21]. The presence of the Fc region in the antibody increases the half-life in the bloodstream, thereby increasing the chances of inducing undesirable auto-reactive immune responses. Aptamers, on the other hand, are smaller molecules with higher tissue penetration rates, but also with a shorter half-life in the blood. The half-life of aptamers ranges from a few hours to one or two days, depending on the aptamer modifications, which is enough time to reach the target and elicit the immunostimulatory effect, but, more importantly, it allows for better managing in case of side effects. Upon the discontinuation of the treatment, the aptamer will be cleared out from blood quickly. In the case of the induction of cytokine storms, such as the CD28 super-agonistic antibody [20], the activity of the aptamer can be easily neutralized within minutes of injection of a universal antidote [22].

## 3. Immune-Checkpoint Antagonizing Aptamers

The induction and maintenance of the immune response is tightly regulated by constant immune checkpoints. Currently, the most relevant immune checkpoints in the field of cancer immunotherapy are Cytotoxic T-Lymphocyte Associated protein 4 (CTLA-4) and Programmed cell Death protein 1 (PD1), which are expressed on the surface of T lymphocytes. The FDA-approved Ipilimumab (anti-CTLA4), Nivolumab, and Pembrolizumab (anti-PD1) monoclonal antibodies are able to unleash the antitumor immune response in cancer patients, showing unprecedented responses in aggressive tumors [23]. We are probably just scratching the surface of the potential of cancer immunotherapy, since there are other interesting immune-checkpoint receptors being tested (T-cell Immunoglobulin and Mucin-domain containing-3 (TIM3), Lymphocyte Activating 3 (Lag3), B- and T-Lymphocyte Attenuator (BTLA), Adenosine A2A Receptor (A2AR), etc.) [24]. However, there are also an increasing number of intracellular immune checkpoints (Forkhead box protein P3 (Foxp3), Signal Transducer and Activator of Transcription 3 (STAT3), Casitas B-lineage Lymphoma-b (cbl-b), Early growth response proteins-2 (EGR-2), Src Homology region 2 domain-containing Phosphatase-1 (SHP1), Src Homology region 2 domain-containing Phosphatase-2 (SHP2), etc.) which are harder to be druggable [25,26,27,28,29,30,31,32,33,34].

CTLA4 is highly homologous to the co-stimulatory receptor CD28; both receptors compete for the same ligands CD80 and CD86, but CTLA4 has a much higher affinity. CTLA4 blockade would probably reduce the threshold of T-cell activation, perhaps favoring the priming of tumor-reactive T cells [35]. CTLA4 also seems to play an important role on Treg function [35]. CTLA4 aptamer was the first immune-modulatory aptamer described. It displayed a high affinity (constant of dissociation (Kd) 30 nM) to CTLA4 and much lower to CD28 (>1 μM) [36]. The aptamer was selected after nine rounds from a 2′F-RNA library of 80 nucleotides against the recombinant murine protein CTLA4-Fc; the size of the aptamer was quite large and was truncated to 35 nucleotides, conserving a similar binding affinity. Furthermore, the aptamer was complexed into a tetramer to be tested in vivo in a therapeutic setting. The CTLA4 tetrameric aptamer showed similar antitumor effects to that of the anti-CTLA4 monoclonal antibody; still, the fact the aptamer needed to be tretramerized to have a therapeutic effect indicates that a better blockade aptamer against mouse and human CTLA4 should be selected [36].

On the other hand, PD1 is one of the main receptors expressed in exhausted T cells. An anti-PD1 DNA aptamer has been recently described. In this case, the aptamer exhibited a more modest Kd (167 nM), but was still enough to block PD1-PDL1 interaction and showed a potent antitumor effect in the PEGylated form in a colon carcinoma murine model [37]. Polyethylene Glycol (PEG) increases the size of the aptamer, which would favor the blocking efficiency and the half-life of the aptamer in blood.

Recently my team has developed the first antagonist TIM3 aptamer. TIM3 is another receptor expressed in exhausted T cells; the blockade of PD1/ Programmed death-ligand 1 (PDL1) and TIM3 has been shown to display a synergistic antitumor effect [38]. The aptamer selected by HT-SELEX had an affinity of 22 nM and was able to block TIM3, enhancing T-cell activation and reducing the tumor load in a colon carcinoma CT26 orthotropic tumor model in combination with anti-PDL1 antibodies [39].

There are also immunosuppressive cytokines secreted in the tumor microenvironment that are recognized by the tumor-infiltrated lymphocyte acting as immune checkpoint. An example of that is IL10. A 2′F-RNA aptamer that blocks the IL10 receptor on the T lymphocyte was described; the aptamer was truncated and selectively modified with 2′-*O*-methyl groups, conserving its activity. The aptamer was tested in vivo eliciting a therapeutic effect in tumor-bearing mice [40].

Immune checkpoints are not only limited to extracellular receptors; there are also very important ones intracellularly. Aptamers can be used to deliver specific cargos (chemotherapy drugs, siRNA, mirRNA, saRNA, peptides, etc.) to the intracellular compartment by targeting cell-specific receptors [41,42,43,44,45,46,47]. So far, the most extended use of this type of reagents is known as aptamer-siRNA chimeras, firstly described by Paloma Giangrande’s group [41,48]. Using a similar approach, Kortylewski et al. generated a CpG-siRNA chimera against STAT-3 transcription factor. STAT-3 is a transcription factor that orchestrates an important part of the tumor immunosuppressive microenvironment in different ways [49]. cytosine–phosphate–guanine enriched oligo (CpG) acts as a natural aptamer binding to Toll Like Receptor 9 (TLR9) expressed in myeloid-derived suppressive cells, macrophages and several types of tumors; the STAT3 siRNA attached to the TLR9 ligand is released in the cytosol, knocking down STAT-3 in the target cell. This approach elicited a potent antitumor immune response capable of rejecting different types of tumors in mouse models [50,51,52]. Recently the CTLA4 aptamer previously described was used to generate a CTLA4 aptamer-siRNA chimera against STAT3 to target the knockdown of STAT3 in tumor-infiltrated T lymphocytes [53]. Mechanistic Target of Rapamycin (mTOR) has recently been underscored as the key checkpoint in immune-memory induction; the expression of mTOR reduces the differentiation of effector cells into T-memory cells. Rapamacyn has been used to inhibit mTOR and enhance the memory T-cell differentiation, but mTOR is also expressed in other cells, such as dendritic cells, where the inhibition hampers the induction of the immune response acting as an immunosuppressive drug. Gilboa’s group circumvented this caveat by using 4-1BB aptamer-siRNA chimera against mTOR (Raptor), where the 4-1BB aptamer targets activated T cells delivering the Raptor siRNA into the cell cytoplasm upon the internalization of 4-1BB (CD137); the siRNA within the cell inhibits mTOR favoring the induction of memory T cells [54].

Another very important intracellular immune checkpoint that regulates the function of Treg is Foxp3 [25,27]. We managed to generate a first-in-class aptamer-peptide chimera to target the inhibition of Foxp3 in T cells. A peptide (P60) that binds to Foxp3, blocking its activity, was previously selected by phage display [25]. The P60 peptide was chemically conjugated with the CD28 aptamer, a receptor that is expressed on T lymphocytes. The aptamer-P60 chimera binds to CD28-expressing lymphocytes inhibiting Foxp3 in the T-cell compartment and enhancing the immune response elicited by a tumor-antigen vaccination [45]. P60 is a peptide that has been described by Casares et al. that can be internalized into the cytoplasm, hijacking Foxp3 in the cytosol, which abrogates Foxp3 translocation into the nucleus [25]. The amount of free P60 peptide that is needed to have a therapeutic effect in vivo is too high. However the conjugation of P60 with the CD28 aptamer favors the accumulation of the P60 peptide in T lymphocytes, improving the efficacy and eliciting a potent antitumor immune response upon tumor antigen vaccination [45].

## 4. Immunostimulatory Aptamers

**Figure 1:**
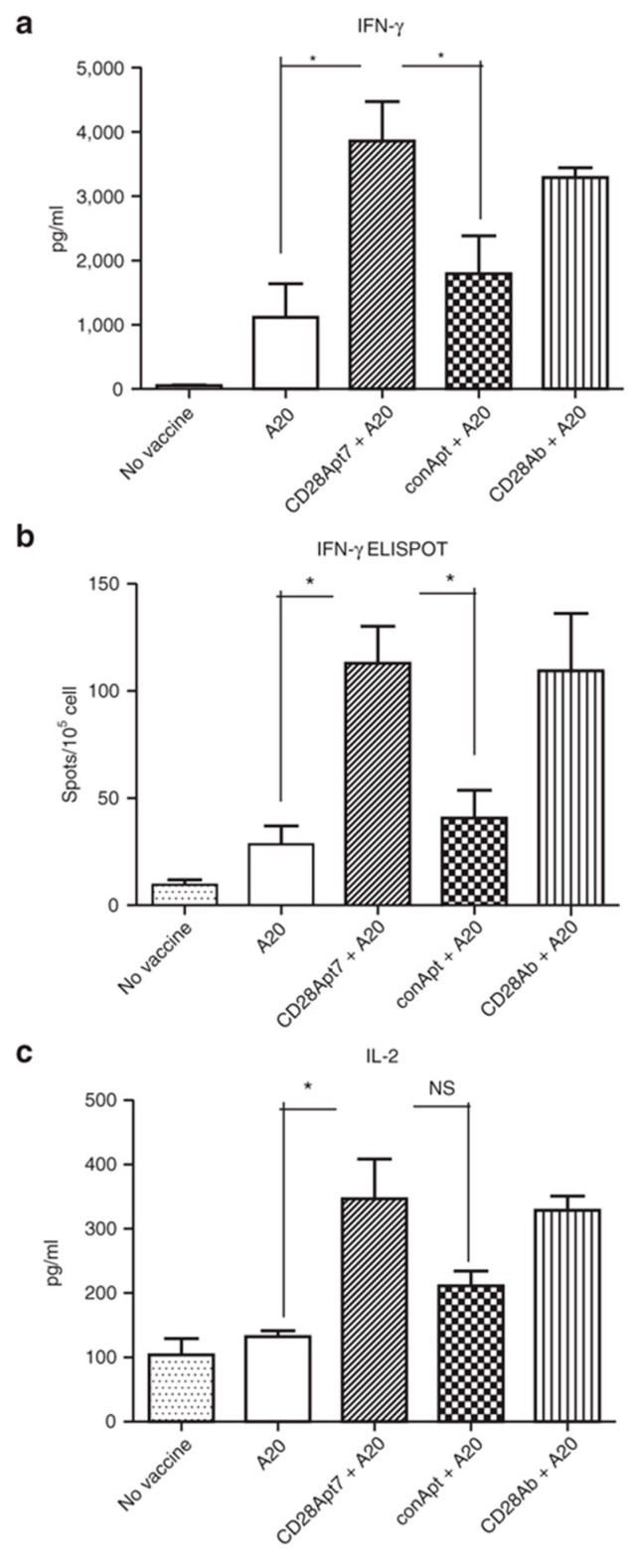
Boosting cellular immune response through the CD28Apt7 dimer. (**a**) Detection of IFN-γ by Enzyme-Linked ImmunoSorbent Assay (ELISA) from supernatant obtained after a 48 h co-culture of irradiated A20 cells and lymphocytes obtained from mice previously immunized with irradiated A20 cells plus 400 pmol of the CD28Apt7 dimer, or CD28 agonistic antibody 37.51, or a scramble aptamer. The results are expressed as mean and SEM of triplicate experiments. (**b**) Detection of IFN-γ-producing lymphocytes through Enzyme-Linked ImmunoSpot Assay (ELISpot) after 24 h co-culture of irradiated A20 cells and lymphocytes obtained from mice previously immunized with irradiated A20 cells plus 400 pmol of the CD28Apt7 dimer, or CD28 agonistic antibody 37.51, or a scramble aptamer. The results are expressed as mean and SEM of triplicate experiments. (**c**) Detection of IL-2 by ELISA from supernatant obtained after a 48 h co-culture of irradiated A20 cells and lymphocytes obtained from mice previously immunized with irradiated A20 cells plus 400 pmol of the CD28Apt7 dimer, or CD28 agonistic antibody 37.51, or a scramble aptamer. The results are expressed as mean and SEM of triplicate experiments. * *p* < 0.05. IFN, interferon; IL, interleukin; NS, not significant. Reproduced from Pastor et al. [60].

Another cancer immunotherapy approach to boost the antitumor immune response is to provide artificial stimulatory or co-stimulatory ligands to tumor antigen-specific lymphocytes [55]. Agonistic antibodies with this type of activity have been around for several years; however, the use of agonistic aptamers was pioneered by Eli Gilboa not long ago [56]. Most of the co-stimulatory receptors expressed in leukocytes trigger the activation signal by crosslinking the intracellular domain of the receptor, so the receptors need to be brought together in order to induce the activation signal. Monoclonal antibodies are dimers that favor the crosslinking of two receptors, which is enough to drive the activation signal. Based on that concept, the first agonistic aptamer was designed as a dimer. The aptamer was selected against the murine 4-1BB co-stimulatory receptor by regular SELEX and dimerized by hybridization of 21-nucleotide complementary sequence extended at the 3’ end of the aptamer. The 4-1BB agonistic aptamer was able to induce the co-stimulatory signal in CD8 lymphocytes measured by proliferation and IFN-γ production with a suboptimal stimulus with anti-CD3 antibody. The aptamer was further evaluated for its antitumor effect in a murine mastocytoma tumor model, showing a similar antitumor effect to that of the 4-1BB agonistic antibody (3H3) [56]. This 4-1BB aptamer was further used in combination with other approaches that are able to induce tumor immunity, such as target inhibition of nonsense-mediated mRNA decay (NMD) [57], or tumor radiation [58]. Several other agonistic aptamers generated as dimers against co-stimulatory receptors have been described, such as OX40 (CD134), CD28, and CD40; these aptamers showed a similar stimulatory effect to that of the equivalent monoclonal antibody in vitro or in vivo [59,60,61] (Figure 1). The type of dimerization has been designed in different ways; for instance, a chemical oligonucleotide scaffold was used for the OX40 agonistic aptamer [59]. Two different types of linkers have been used for the CD28 aptamer, displaying different co-stimulatory activities, indicating that the length, orientation, and probably the flexibility could affect the capacity of inducing a more or less efficient crosslink of the receptor [60]. It is very likely these aptamers could even trigger a more potent stimulatory signal with a higher level of multimerization, utilizing approaches that have been previously described for other applications [62].

However, with these types of reagents we are at high risk of triggering important side effects, as they would activate indiscriminately all of the lymphocytes in the body that are expressing the co-stimulatory receptor. That could have very serious deleterious effects, as it was observed with the superagonist CD28 antibody clinical trial in which several patients suffered massive cytokine storms [20]. 4-1BB agonistic antibody also induced autoimmune-like hepatitis with high lymphocyte infiltration, splenomegaly, and neutropenia [63]. A possible way to palliate the side effects of this type of treatments is to target the co-stimulation only to the tumor-antigen-specific lymphocytes, which can be achieved by using bi-specific aptamers that, on part aptamer is able to interact with a molecule or receptor that would be expressed only in the tumor, while the other one provides the interaction with the co-stimulatory receptor triggering its activation. This approach, as a proof of concept, was first described to target the surface receptor Prostate-Specific Membrane Antigen (PSMA) expressed on tumor cells for which there was a high affinity aptamer available [64]. For this type of approach it is desirable to allow the bi-specific aptamer to stay in the cell surface as long as possible. PSMA belong to a minority type of receptors that internalize by clathrin-coated pits, so the receptor was modified to reduce its internalization removing the clathrin-binding domain (ΔPSMA). The PSMA aptamer was attached to the 4-1BB agonistic aptamer; both parts of the aptamers were functional, as they were able to bind to PSMA cells and induce 4-1BB co-stimulation on CD8 lymphocytes. The bi-specific aptamer was able to elicit an antitumor immune response dependent on 4-1BB, as the blockade with the 4-1BB-Fc recombinant protein abrogates CD8 infiltration in the tumor. Therefore, the PSMA-4-1BB aptamer was able to increase lymphocyte infiltration in the tumor, but we did not prove whether that was the result of new lymphocytes homing to tumors driven by the bi-specific aptamer, or of in situ proliferation of already-infiltrated lymphocytes; those would be interesting studies to be done in the future. The bi-specific aptamer was able to home only to ΔPSMA-expressing tumors, potentiating the immune response especially in ΔPSMA-expressing tumors in early stages of treatment.

The major advantage of this approach is that we were able to reduce the effective therapeutic dose over the non-targeting agents very significantly (aptamer and antibody), which would be translated into lesser toxicity. In fact, we did not observe any of the toxicity associated with the treatment 4-1BB agonistic antibodies. Either the targeting or non-targeting 4-1BB agonistic aptamer at their therapeutic dose did not display the toxicity observed with the 4-1BB antibody; that may be related with the bio-distribution and the higher half-life of the antibody in the blood (likely attributed to the Fc region or isotype). As an extension of this work Schrand et al. developed a bi-specific aptamer to target 4-1BB co-stimulation to the tumor stroma, by using an aptamer against Vascular Endothelial Growth Factor (VEGF), which is a soluble cytokine overexpressed in several types of tumors [65]. This is a very interesting approach as it could be applicable in most types of tumors, allowing for VEGF blockade and triggering T-cell activation simultaneously in the tumor. This targeting approach showed a better therapeutic index than the non-targeting 4-1BB agonistic antibody. We have also recently developed a bi-specific aptamer to target CD28 co-stimulatory aptamers to tumors that overexpressed MRP1, which is a chemotherapy-resistant channel up-regulated in cancer stem cells, cells with higher resistance to chemotherapy [66]. Similar strategies could also be used to target immune-checkpoint blockade aptamers (CTLA4, PD1, TIM3, etc.) to the tumor in order to reduce undesirable immune reactions in other tissues as well. Using this type of construct we were also able to generate the first irradiated whole-cell tumor vaccine decorated with artificial co-stimulatory aptamers. The use of irradiated whole-cell vaccines genetically engineered to express immunostimulatory molecules has shown very promising results in preclinical settings, as they are able to elicit an immune response against the specific tumor antigens expressed in the tumor [67]. However, translation to the clinic is technically challenging, as an autologous genetically-modified tumor cell line for each patient needs to be generated [68]. The use of bi-specific co-stimulatory aptamers that are able to attach to the tumor cell surface would significantly simplify the generation of this type of vaccine; we have coined the term AptVax as the vaccine of tumor-irradiated cells coated with co-stimulatory bi-specific aptamers [66].

Yet, a main limitation factor in cancer immunotherapy is the lack of tumor antigenicity; several tumors do not express enough potent tumor neo-antigens to be recognized by the immune system as a foreign rejectable tissue. The use of immune-checkpoint blockade agents or co-stimulatory agonists on low antigenic tumors would elicit poor antitumor immune responses [69,70,71,72]. We managed to partially overcome this limitation by targeting the inhibition of NMD [57]; the NMD precludes the expression of mutant transcripts that display premature stop codons; therefore, several of the tumor antigens that are not going to be expressed at the desirable concentration to be detected by the immune system could be up-regulated upon the inhibition of NMD. This was achieved by using tumor-targeted siRNA chimeras, inducing the inhibition of the NMD only in the tumor cells and not in other tissues. As it could be expected, the use of 4-1BB co-stimulatory aptamers potentiates the antitumor immune response elicited by the target inhibition of NMD [57].

## 5. Conclusions

**Figure 2:**
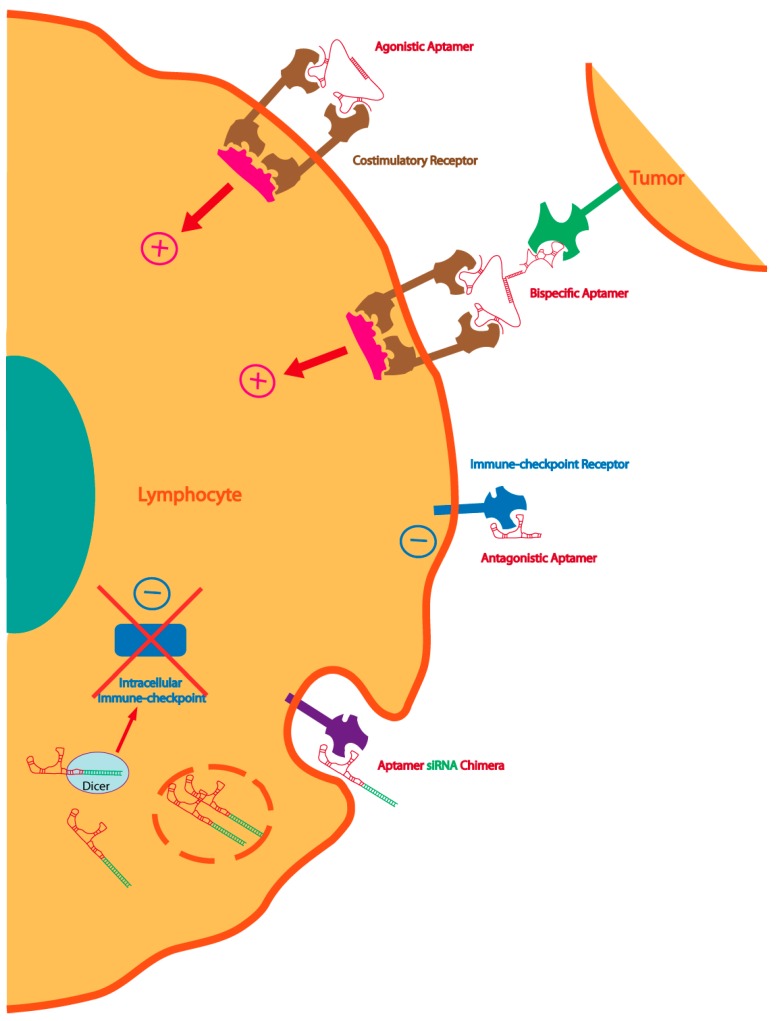
Multiple strategies to enhance immunity with aptamers: dimeric or multimeric aptamers against co-stimulatory receptors can be used as agonistic molecules of the cognate receptor. The same agonistic aptamer can be delivered to the tumor, improving the therapeutic index, by coupling with a tumor-specific aptamer. Immune-checkpoint blockade aptamers against cell-surface receptors can be used to enhance tumor immunity. The blockade of the intracellular immune-checkpoint that orchestrates an immune suppression pathway can be inhibited by targeting aptamer-siRNA chimeras to the lymphocyte or the tumor cell.

Since the selection of the first aptamer by Tuerk et al. in 1990 [9], one aptamer (Macugen) has already reached clinical approval for the treatment of macular degeneration (intravitreal injection). However, the number of aptamers that have reached the clinical pipeline is still not very high. The reason for that could be: (1) Competition with monoclonal antibodies. Monoclonal antibodies are very extensively used in research, while aptamers are still quite unknown. We are, by nature, reluctant to change to new technologies, especially when something is already working; (2) Aptamers have been under a very well protected patent until quite recently, pushing away some of the interest by some companies to get these reagents into the clinic; (3) A recent phase-III clinical trial with REG1 (anti-IX coagulation factor PEGylated aptamer) turned out to be toxic in 10 of 1605 patients (including one fatal reaction). This created a significant amount of disappointment as intravenous injection of aptamers could be toxic in a small fraction of patients, discouraging pharmaceutical companies to get aptamers into clinical trials. However, in a very recent analysis by RADAR (A **R**andomized, Partially-Blinded, Multi-Center, **A**ctive-Controlled, **D**ose-ranging Study **A**ssessing the Safety, Efficacy and Pharmacodynamics of the **R**EG1 Anticoagulation System in Patients with Acute Coronary Syndromes), it turned out to be that the toxicity was due to a PEG allergic reaction and not to aptamers, as the small fraction of patients that show signs of toxicity have anti-PEG antibodies [73,74]. In spite of all of that, in the last few years, aptamers have re-emerged in preclinical settings, and will hopefully account for a significant amount of therapeutic drugs for different applications in future medicine. The use of aptamers in cancer immunotherapy is more recent (Table 1). This versatile technology allows for the tight manipulation of the immune system at different levels, either by targeting different leukocyte subtypes or the tumor. Not only can aptamers be used as antagonists or agonists of an accessible receptor, but they can also be used for the delivery of specific cargos, such as siRNA, to inhibit pathways in the target cell, or saRNA, to activate the expression of a gene, or even other aptamers [42,48,64] (Figure 2). It is very likely that the awareness of this new therapeutic platform in cancer immunotherapy will bring more interest to the technology, reducing the chemical high-scale cost of production and accelerating its translation to the clinic in the near future.

## Figures and Tables

**Table 1:** List of immunomodulatory aptamers described in cancer immunotherapy.

Aptamer	Application in Cancer Immunotherapy	Type of Tumor
CTLA-4	Immune-checkpoint blockade [36]	Melanoma
	Targeting STAT3 siRNA [53]	Lymphoma, Colon Cancer, Kidney Cancer, Fibrosarcoma
PD1	Immune-checkpoint blockade [37]	Colon Cancer
TIM3	Immune-checkpoint blockade [39]	Colon Cancer
IL10R	Immune-checkpoint blockade [40]	Colon Cancer
4-1BB	Costimulatory receptor agonist [56]	Mastocytoma
	Targeting costimulation to the tumor [64,65]	Melanoma, Colon Cancer, Breast Cancer, Oncogene-induced high-grade Glioma, MCA Fibrosarcomas
OX40	Costimulatory receptor agonist [59]	Melanoma
CD28	Costimulatory receptor agonist [60]	Lymphoma
	Targeting costimulation to the tumor [66]	Melanoma
CD40	Stimulatory receptor agonist [61]	Lymphoma
